# Fractures around Trochanteric Nails: The “Vergilius Classification System”

**DOI:** 10.1155/2021/7532583

**Published:** 2021-01-12

**Authors:** Giuseppe Toro, Antimo Moretti, Daniele Ambrosio, Raffaele Pezzella, Annalisa De Cicco, Giovanni Landi, Nicola Tammaro, Pasquale Florio, Antonio Benedetto Cecere, Adriano Braile, Antonio Medici, Antonio Siano, Bruno Di Maggio, Giampiero Calabrò, Nicola Gagliardo, Ciro Di Fino, Gaetano Bruno, Achille Pellegrino, Giacomo Negri, Vincenzo Monaco, Michele Gison, Antonio Toro, Alfredo Schiavone Panni, Umberto Tarantino, Giovanni Iolascon

**Affiliations:** ^1^Department of Medical and Surgical Specialties and Dentistry, University of Campania “Luigi Vanvitelli”, Naples, Italy; ^2^Department of Clinical Sciences and Translational Medicine, University of Rome Tor Vergata, Rome, Italy; ^3^Unit of Orthopaedics and Traumatology, Evangelical Hospital Betania, Naples, Italy; ^4^Department of Life Health & Environmental Sciences, University of L'Aquila, Unit of Orthopaedics and Traumatology, L'Aquila, Italy; ^5^Unit of Orthopaedics and Traumatology, AORN S. Giuseppe Moscati, Avellino, Italy; ^6^Unit of Orthopaedics and Traumatology, Santa Maria Della Speranza Hospital, Battipaglia, Italy; ^7^Unit of Orthopaedics and Traumatology, “Ave Gratia Plena” Civil Hospital, Piedimonte Matese, Italy; ^8^Unit of Orthopaedics and Traumatology, San Francesco D'Assisi Hospital, Oliveto Citra, Italy; ^9^Unit of Orthopaedics and Traumatology, San Giuliano Hospital, Giugliano, Italy; ^10^Unit of Orthopaedics and Traumatology, AOR San Carlo, Potenza, Italy; ^11^Unit of Orthopaedics and Traumatology, AORN Sant'Anna e San Sebastiano, Caserta, Italy; ^12^Unit of Orthopaedics and Traumatology, San Giuseppe Moscati Hospital, Aversa, Italy; ^13^Unit of Orthopaedics and Traumatology, Santa Maria Incoronata Dell'Olmo Hospital, Cava de' Tirreni, Italy; ^14^Unit of Orthopaedics and Traumatology, Villa Malta Hospital, Sarno, Italy

## Abstract

**Introduction:**

The fractures that occurred around trochanteric nails (perinail fractures, PNFs) are becoming a huge challenge for the orthopaedic surgeon. Although presenting some specific critical issues (i.e., patients' outcomes and treatment strategies), these fractures are commonly described within peri-implant ones and their treatment was based on periprosthetic fracture recommendations. The knowledge gap about PNFs leads us to convene a research group with the aim to propose a specific classification system to guide the orthopaedic surgeon in the management of these fractures.

**Materials and Methods:**

A steering committee, identified by two Italian associations of orthopaedic surgeons, conducted a comprehensive literature review on PNFs to identify the unmet needs about this topic. Subsequently, a panel of experts was involved in a consensus meeting proposing a specific classification system and formulated treatment statements for PNFs. *Results and Discussion*. The research group considered four PNF main characteristics for the classification proposal: (1) fracture localization, (2) fracture morphology, (3) fracture fragmentation, and (3) healing status of the previous fracture. An alphanumeric code was included to identify each characteristic, allowing to describe up to 54 categories of PNFs, using a 3- to 4-digit code. The proposal of the consensus-based classification reporting the most relevant aspects for PNF treatment might be a useful tool to guide the orthopaedic surgeon in the appropriate management of these fractures.

## 1. Introduction

Fractures around nails are a huge challenge for the orthopaedic surgeon with a constantly rising incidence due to the increased frequency of hip fragility fractures worldwide [1, 2]. These fractures are commonly classified into two groups “intracapsular” and “extracapsular” (EF). Although considered as a unique entity, these two types differ largely in terms of pathoanatomy, clinics, epidemiology, and management [3]. In this regard, while intracapsular fractures are treated using hemiarthroplasty (HA) or total hip arthroplasty (THA), EF is generally fixed, most commonly, using trochanteric nails (TNs) [4]. However, TNs are associated with some complications, including perinail fractures (PNFs), whose incidence is expected to grow up in the next years [5]. The available evidence on PNFs concerning their clinical and treatment issues is poor and confusing, since these fractures are generally described along with those occurred around femoral plates, and both anterograde and retrogade interlocking nails (namely, peri-implant fractures) [5–7]. Moreover, the treatment of PNFs is often extrapolated by periprosthetic fracture (PPF) management [7, 8]. These latter fractures are generally treated using revision surgery or plate fixation, depending on fracture localization, prosthesis loosening, and bone stock, as defined by the Vancouver classification [9]. However, PNFs present several differences with respect to PPF, including treatment outcomes, particularly higher mortality rate in PNFs [10, 11]. Moreover, in our opinion, Vancouver classification use for PNFs is somewhat questionable and PNFs must be considered more appropriately a unique entity [7]. The growing interest in these fractures led some authors to propose other classifications of peri-implant fractures [5–7]. To the best of our knowledge, there is a gap in classifying PNFs and, consequently, guiding their treatment. This unmet need has driven us in a stepwise procedure aiming to propose a specific classification system and a practical guide for the treatment of PNFs.

## 2. Materials and Methods

The present study was the first of a three-step protocol based on (1) a consensus conference aimed to identify the fracture characteristics considered relevant for their classification and management; (2) retrospective multicentre observational study to apply the new classification system to our patients with PNFs; and (3) a position statement aimed to define the most appropriate PNFs treatment protocol. This kind of approach was recommended by Audigé et al. [12] and has been widely used for the proposal of new classifications [13].

In this first study, a steering committee, consisting of 7 members (5 orthopaedic surgeons with expertise in fragility fractures and 2 clinical researchers with expertise in performing systematic reviews), identified by the Italian group for the study of severe osteoporosis (*Gruppo Italiano per lo studio dell'osteoporosi severa, G. I. S. O. O. S.*) and the Association of Orthopaedics and Traumatologists of Campania (*Associazione Campana Ortopedici e Traumatologi Ospedalieri, A. C. O. T. O.*), conducted a comprehensive literature review in October 2018. The steering committee identified key evidence on the diagnosis and treatment of PNFs. The systematic research was conducted in MEDLINE and EMBASE, using different combinations of keywords (i.e., peri nail fractures AND diagnosis; peri implant fractures AND diagnosis; peri prosthetic fracture AND diagnosis; trochanteric nail AND fracture AND diagnosis; peri nail fractures AND treatment; peri implant fractures AND treatment; peri prosthetic fracture AND treatment; trochanteric nail AND fracture AND treatment) between January 2000 and November 2018.

All articles in English, Italian, and Spanish languages were included.

The committee selected the most relevant papers among those identified, based on the following inclusion criteria:

1. Articles on femoral peri-implant fractures
2. Articles on surgical techniques and related outcomes on periprosthetic Vancouver B1 and C fractures
3. Articles on surgical techniques and related outcomes on interprosthetic and/or interimplant femoral fractures
4. Articles on trochanteric nail complications (excluding those designed on specific complications like the cutout)

Articles on periprosthetic knee femoral fractures were also excluded.

After the literature research, the steering committee defined some questions concerning current unmet needs in the management of PNFs during their first meeting in January 2019.

The open issues identified by the steering committee were as follows:

1. PNF definition
2. PNF management
3. PNF classification

**Figure 1:**
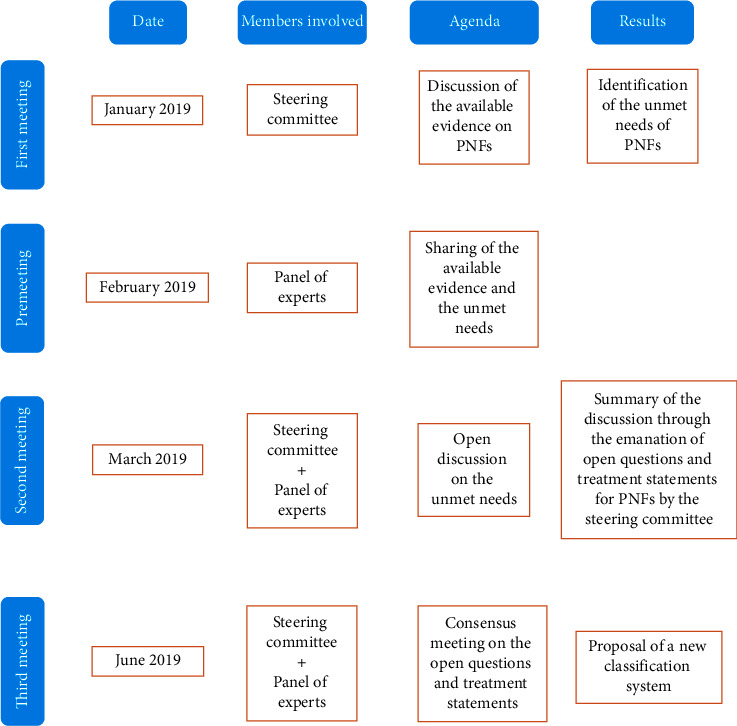
Summary of the entire procedure done to obtain the consensus for the classification system.

These open issues were proposed to a panel of 18 orthopaedics with specific expertise in geriatric orthopaedics and trauma surgery, during two further meetings. The entire procedure through which the present study was conducted is summarized in Figure 1.

During the last meeting, the steering committee asked the panel of experts to give their consensus on the open questions and treatment statements on PNFs. During this meeting, the research group decided that all the characteristics of PNFs considered relevant for their treatment would be included in a specific classification system. A secret voting session was performed, and the relevant characteristics that got more than 65% of votes would have been included in the classification proposal. The classification categories were identified in hierarchical order according to the grade of consensus. Finally, the classification proposal was approved by both steering committee and panel members.

## 3. Results and Discussion

**Figure 2:**
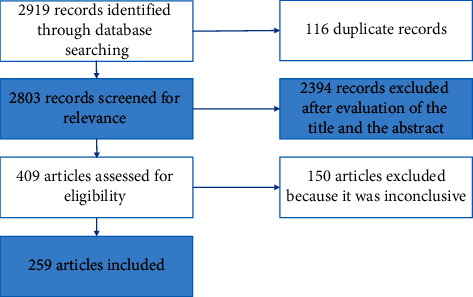
Research strategy.

2919 articles were analysed after the first literature research. After title and abstract revision and duplicates removal, 2510 articles were excluded. Further 150 articles were excluded after full-text reading because it was considered inconclusive for the main objective of the study. Therefore, 259 articles were included and discussed by the steering committee during their first meeting (Figure 2).

The answers to each open question are summarized in Table 1, while the statements proposed for the management of PNFs are shown in Table 2.

According to the research group (namely, the steering committee and the panel of expert), the definition of PNF is “a fracture occurred around or next to a TN”.

The research group included four PNF characteristics considered relevant for their treatment in the classification proposal in the following hierarchical order:

1. Fracture localization (around the nail (namely, from the cephalic to the distal screw), around the distal screw, and distal from the tip of the nail), consensus 98%
2. Fracture morphology (spiral, oblique, and transverse), as defined by AO [14], consensus 98%
3. Fracture fragmentation (2, 3, or more than 3 fracture fragments), consensus 92%
4. Healing status of the previous EF (healed or not healed), consensus 92%

The type of the implanted nail was not considered relevant, whereas no consensus was obtained regarding the nail length.

**Figure 3:**
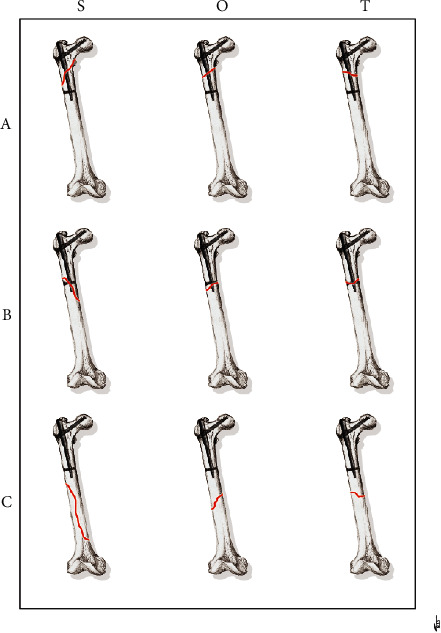
The main 9 types of PNFs.

**Figure 4:**
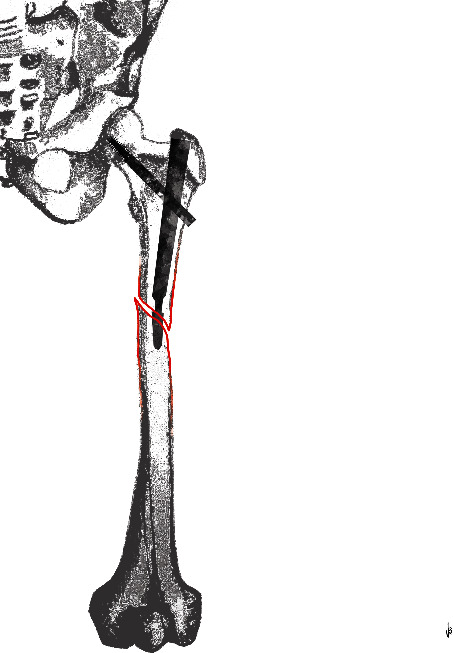
A drawing representing a BO2 fracture.

**Figure 5:**
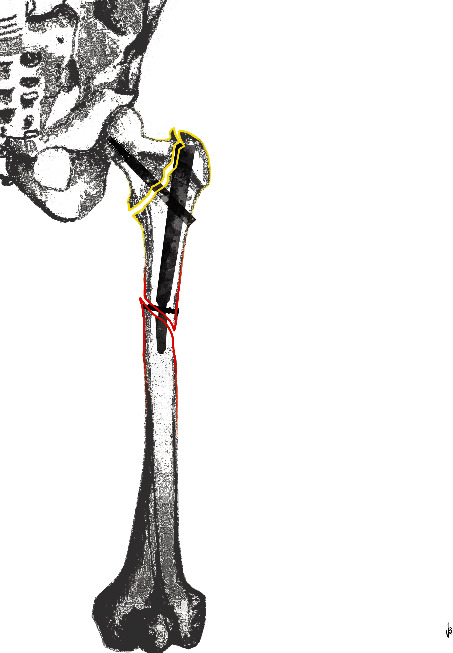
A drawing representing a BO2n fracture.

An alphanumeric code was proposed to identify each characteristic: (A) fracture around the nail; (B) fracture around the distal screw; (C) fracture distal from the tip of the nail; (S) spiral fracture; (O) oblique fracture; (T) transverse fracture; (2) two fracture fragments; (3) three fracture fragments; (3+) more than three fracture fragments; and (n) EF not healed. Therefore, a total of 54 potential categories were proposed in our classification and a single fracture would be described by 3 to 4 codes (see Figures 3–5).

Our study has the main purpose of covering a gap in the knowledge of PNF management, proposing, through a scientifically corroborated procedure, an algorithm for treating it. This gap concerns, above all, an appropriate classification of PNFs since those available were considered inadequate as treatment guide by our research group and led us to propose a new classification system from which some statements concerning specific management indications are derived.

PNFs (fractures occurred around or next to an implanted TN) have been a challenge for the orthopaedic surgeon. Some characteristics (fracture localization, fracture morphology, fracture fragmentation, and healing status of the previous fracture) were considered relevant for their management.

The relevance of PNFs was demonstrated by the growing literature around this topic [5–8, 15–17] and was justified by the expected increase in PNF incidence due to the rise of osteoporotic fractures [1, 2].

Osteoporosis is considered one of the main health problems in developed countries, considering the high morbidity and mortality of fragility fractures and the resulting economic burden [18]. The proximal femur is one of the most affected sites by fragility fractures [1].

Proximal femoral fractures are classified into intracapsular and EF depending on the anatomic localization of the fracture line. Although several studies reported that a clear difference did not exist between the plate and TNs for the treatment of EFs [19], these latter devices are the preferred fixation methods by the orthopaedic surgeons, probably because of their supposed easy-to-use and minimally invasive technique [4, 20]. In a recent epidemiological study on EF management in Sweden, over 6,000 nails were used to treat 10548 fractures [21]. However, some implant-related complications were frequently reported including cutting out, cut through, secondary fracture displacement, and PNFs [22].

These were an emerging complication of TN fixation with high mortality and morbidity [15], and their incidence was reported to be around 2% [16, 23].

Generally, PNFs were treated using the recommendation of PPF [7]. However, several differences could be seen in terms of both technical issues and patient outcomes comparing PNFs and PPF [10, 11]. For example, the former demonstrated a 1-year mortality rate higher than PPF (21% vs. 13%) [10, 11]. Moreover, factors that guide the treatment decision making of PPF (i.e., prosthesis stability and bone loss) could not be used for PNFs.

According to our classification, fracture localization, fracture morphology, fracture fragmentation, and healing status of the previous EF were the most relevant factors for the management of PNFs. The fracture localization was considered relevant because fixation devices are anatomically shaped (i.e., distal femur plates are specifically designed for the distal part of the femur) and the choice of a specific fixation device depends also on the relationship of the fracture line with the implanted TN. However, for 1 of the members of the panel, the definition of B type (around the distal screw) should be more appropriately as “parting from the distal screw”; nevertheless, during the discussion, the vast majority of the members agreed with the proposal of the steering committee, considered less confusing.

One of the members does not consider relevant fracture morphology because in his experience all fractures were oblique. Moreover, two other members of the research group questioned the relevance of fracture fragmentation because, in their personal experience, most of the PNFs were simple 2 fragment fractures. However, most of the members of the research group were in agreement that fracture morphology and fragmentation must be considered relevant because they affect primary fracture stability [24, 25] and guide the fixation technique (i.e., the choice between relative or absolute stability) [26]. For example, the intrinsic stability and the difficulty in obtaining nail-periosteum contact in femoral spiral fractures make mandatory the use of locked nails or plates [24]. However, the relevance of the aforementioned factors was generally known, considering that these were at the base of the AO classification of bone fractures since its first edition [27].

The healing status of the previous EF might complicate the fracture pattern, resembling an ipsilateral femoral neck and shaft fracture. These considerations were not accepted by two research members, especially regarding the choice of the fixation device, because several reports proposed the use of a single fixation device (namely the nail) for the treatment of ipsilateral femoral neck and shaft fractures [28, 29].

The aforementioned relevant factors were in line with some reported in the previously published classifications [6, 7]. In fact, while Skela-Rosenbaum et al. considered fracture localization (from proximal to distal) and morphology (spiral fractures) as a guide for the treatment decision making [6], the healing status of the previous fracture was considered relevant by Chan et al. [7].

Other proposed factors were not part of the classification proposal since the research group did not consider the type of implant (monoaxial and biaxial) a pivotal factor for the management of PNFs. The difference between a monoaxial and a biaxial TN depends on the number of pins, screws, or blades that cross the fracture line [16]. In fact, while the biaxial TN presents two screws, pins, or blades that cross the fracture line, the monoxial just one. Norris et al. demonstrated that biaxial TNs were associated with lower fracture risk compared with monoaxial ones [16]. This could be linked to the lower positioning of the lower screw of the biaxial TN that allows a reduction in the stress concentration on the proximal femur [16]. However, in our opinion, this supposed difference in the pathogenesis of PNFs did not affect the treatment decision making.

The relevance of nail length in determining PNFs is still a matter of debate [30–32]. Recently, Shannon et al. in their randomized controlled trial reported a similar rate of PNFs around both short and long nails (2.4% vs. 2.7%) [30]. Moreover, Daner et al. in their biomechanical study did not observe any difference in stiffness between long and short nails under rotational forces, thus making the fracture risk similar [17]. These inconclusive evidences on the role of nail length in determining PNFs and the observation that a clear definition of long nail did not exist (long nails are considered those longer than 240 mm, 300 mm, and 340 mm depending on the nail and the authors [32–35]) did not lead the research group to reach a consensus on the putative role played by nail length in their PNF management.

Our classification proposal was not the first on femoral peri-implant fractures [5–7]. Interestingly, all these classifications have been published in the last four years, underlying the relevance of the topic and the need for treatment guidance. However, in our opinion, none of the previously published classifications was able to accurately describe the PNF pattern and to guide treatment decision making (see Table 3 for further details).

The classification proposal by Videla-Cés et al. was performed by studying 143 peri-implant fractures. The classification extrapolated some concepts from the AO/OTA classification (i.e., the alphanumeric code). Their classification aimed to morphologically describe all peri-implant femoral fractures, giving information on fracture localization (from 1 to 3), the type of implant used (N for nails, P for plates, and D for retrograde implants), and fracture relationship with the implant (from A to E). In the classification proposed by Videla-Cés et al., fracture fragmentation or previous fracture healing (except for type E) was not considered. Although the comprehensive anatomical and morphological description was made by this classification, the lack of some relevant factors (fracture fragmentation and healing status of the previous fracture) reduced its applicability for treatment purpose.

Chan et al., instead, classified peri-implant fractures according to the type of implant (N for nail and P for plate), fracture localization (at the tip of the implant or distal from the implant), and healing status of the previous fracture. Their classification proposal hereby authorised to classify peri-implant fractures regardless of the bone affected, but they performed an extreme simplification of the fracture characterization, excluding several types of fractures (i.e., fracture around the implant and comminuted fractures).

Skela-Rosenbaum et al., evaluating a series of 21 PNFs, proposed a simple classification system based on three types and two subtypes (IA trochanteric metaphyseal, IB proximal metadiaphyseal; IIA fracture at the tip of the nail, IIB fractures extending from the tip of the nail; and III fractures of the distal femur). The main factors evaluated in their classification were fracture localization and morphology. However, the classification is purely descriptive, and it was impossible to combine the factors included (i.e., it is not possible to classify a spiral fracture occurred around the nail). Moreover, in their classification proposal, the fragmentation and the healing status of the previous fracture were not evaluated. These factors represented a limit for an appropriate fracture characterization, reducing the applicability of their classification as a treatment guide only to some specific types of PNFs.

When proposing a classification system, it should be reliable, simple to use, and serve as the basis for treatment [36]. In these perspectives, we choose a simple alphanumerical code also exploiting some of the codes used in well-known classifications of the orthopaedic world. In fact, the use of the ABC code for the fracture localization was the same as the Vancouver classification of PPF [9]. Instead, we preferred the SOT code to describe the fracture morphology using the first letter of the words “spiroid”, “oblique,” and “transverse”. In a similar way, the fragmentation was encoded using the number of fragments observed (2, 3, and +3). Lastly, the nonhealing status was coded adding an “n” at the end of the alphanumeric code. Therefore, a two-fragment oblique fracture occurred around the distal screw of a TN would be classified as B (around the distal screw) O (oblique fracture) 2 (see Figure 4). In case the previous EF was not healed yet, then the same fracture should be classified as BO2n (see Figure 5).

Our classification proposal, based on expert opinions, included 54 possible categories of PNFs, to characterize them considering fracture localization, morphology, and fragmentation and healing status of the previous fracture. These factors are all considered relevant for the management of PNFs; therefore, this new classification system fulfils the intended purpose of providing a simple tool for guiding treatment choice for PNFs. However, before its applicability, a cohort study is required.

## 4. Conclusions

The growing incidence of proximal femoral fractures and the large use of TN will lead to a constant increase of PNFs. However, their diagnosis is still underrated, considering that they are generally included along with fractures occurred around plates in a generic “peri-implant” fracture category. However, PNFs are a separate entity with high patients' morbidity and mortality that needs specific strategies to be treated. The proposal of a simple classification based on the most relevant aspects for fracture treatment might be a useful tool to guide the orthopaedic surgeon. Moreover, the use of a specific classification system would be beneficial also to standardize the literature terminology to improve the evidence around PFNs. However, case-based studies are needed to validate the present classification before its widespread use.

## Figures and Tables

**Table 1:** Open question answers and grade of consensus.

Question	Answer	Grade of consensus (%)
Is the definition of perinail fracture a fracture occurred around or next to an implanted nail?	Yes	100
Is fracture localization (around the nail (namely, from the cephalic to the distal screw), around the distal screw, distal from the tip of the nail) a pivotal factor for PNF management?	Yes	98
Is fracture morphology (spiral, oblique, and transverse), as defined by AO, a pivotal factor for PNF management?	Yes	98
Is the fracture fragmentation (number of fragments (2, 3, and more than 3 fragments)) a pivotal factor for PNF management?	Yes	92
Is the healing status of the previous EF (healed or not healed) a pivotal factor for PNF management?	Yes	92
Is the type of implanted TN (namely, biaxial or monoaxial) a pivotal factor for PNF management?	No	100
Is TN length (short or long using 300 mm as a threshold) a pivotal factor for PNF management?	Yes	50
Do you think that the currently available classification systems are useful for the management of PNFs?	No	100

**Table 2:** Statements on the management of PNFs.

Statement	Description
Statement #1	Fracture localization is a pivotal factor for PNF management. The fixation device used depends largely on the part of the femur involved by the fracture line and its relationship with the implanted nail.
Statement #2	Fracture morphology is a pivotal factor for PNF management, considering the fracture intrinsic stability. The description of fracture line orientation made by AO is appropriate for the definition of PNF morphology.
Statement #3	Fracture fragmentation is a pivotal factor for PNF management.
Statement #4	The healing status of the previous EF is a pivotal factor for PNF management as it can significantly reduce treatment options.
Statement #5	The type of implanted TN is not a pivotal factor for PNF management.
Statement #6	TN length may affect the fracture pattern, but it is unclear if it acts on PNF management.
Statement #7	The available classification systems are not useful for guiding the treatment decision making of PNFs

**Table 3:** Published peri-implant femoral fracture classifications.

Authors	Journal	Year of publication	Pros	Limitation	Main differences with the present classification
Skela-Rosenbaum et al.	Injury	2016	Treatment-oriented classification; classification included only PNFs; evaluated fracture occurred around a single type of nail	Not validated; did not consider the original fracture healing status; classified fracture occurred only around short nails	Original EF healing status considered; classification of fracture occurred around long nails; comprehensive morphological classification; evaluation of fracture fragmentation
Chan et al.	Arch Orthop Trauma Surg	2017	Treatment-oriented; evaluated the original EF healing state	Included also fractures occurred around plates; did not include fracture occurred in the proximal part of the nail; did not consider fracture fragmentation and morphology	Comprehensive morphological and anatomical classification; classification of fracture occurred only around TN; evaluation of fracture fragmentation
Videla-Cés et al.	Injury	2018	Large cohort, comprehensive anatomical and morphological classification	Not treatment-oriented; included also fractures around plates; not validated; did not consider the original fracture healing status; did not consider fracture fragmentation	Original EF healing status considered; fracture fragmentation considered; classification of fracture occurred only around TN

## Data Availability

The data from articles supporting this consensus conference are from previously reported studies and datasets, which have been cited. The processed data are available from the corresponding author upon request.
